# Phosphorylated EGFR (pEGFR T693) as a Novel Predictor of Recurrence in Non-Functioning Pituitary Adenomas

**DOI:** 10.3389/fendo.2021.708111

**Published:** 2021-07-05

**Authors:** Ashutosh Rai, Liza Das, Kanchan K. Mukherjee, Sivashanmugam Dhandapani, Manjul Tripathi, Chirag Kamal Ahuja, Bishan Dass Radotra, Pinaki Dutta

**Affiliations:** ^1^ Department of Endocrinology, Postgraduate Institute of Medical Education and Research (PGIMER), Chandigarh, India; ^2^ Department of Neurosurgery, Postgraduate Institute of Medical Education and Research (PGIMER), Chandigarh, India; ^3^ Department of Radiodiagnosis, Postgraduate Institute of Medical Education and Research (PGIMER), Chandigarh, India; ^4^ Department of Histopathology, Postgraduate Institute of Medical Education and Research (PGIMER), Chandigarh, India

**Keywords:** pEGFR T693, non-functioning pituitary adenomas, recurrence, biomarker, prognosis

## Abstract

**Purpose:**

Non-functioning pituitary adenomas (NFPAs) exhibit high recurrence rates after surgery. However, the determinants of recurrence are inconsistent in the available literature. The present study sought to investigate the association between nuclear phosphorylated EGFR (pEGFR) levels and recurrence of NFPAs.

**Methods:**

Tissue microarrays from patients undergoing adenomectomy for NFPAs at our tertiary care center from 2003 to 2015 and having a minimum of 60 months of follow-up (n=102) were accessed. Immunohistochemical analysis (IHC) was performed to determine the expression of nuclear pEGFR T693. h-score was calculated as the product of staining intensity and the number of positively staining cells. Radiological surveillance (MRI) was performed to categorize NFPAs as recurrent or non-recurrent on follow-up.

**Results:**

The mean age of the cohort was 50 ± 11 years with a male preponderance (61.1%). Recurrence was observed in 46.1% of the patients at a median of 123 months (IQR 72-159) of follow-up. pEGFR T693 positivity was higher in a significantly greater number of recurrent NFPAs as compared to non-recurrent NFPAs (95.7% *vs* 81%, p=0.02). h-scores were also significantly higher in recurrent NFPAs (122.1 ± 6 *vs* 81.54 ± 3.3, p<0.0001). pEGFR T693 positivity significantly predicted recurrence in NFPAs (HR=4.9, CI 2.8-8.8, p<0.0001). ROC analysis revealed an h-score cutoff of 89.8 as being associated significantly with recurrence (sensitivity 80%, specificity 78%, AUC 0.84, p<0.0001).

**Conclusion:**

pEGFR T693 was expressed in significantly higher number of recurrent NFPAs. The h-scores were also higher in recurrent NFPAs. Nuclear pEGFR T693 may serve as a predictor of recurrence in NFPAs.

## Introduction

Pituitary adenomas are the third most frequently diagnosed intracranial tumors, accounting for approximately 10–25% of all primary cranial neoplasms (1). The majority of pituitary adenomas exhibit aggressive behavior characterized by rapid cell growth, despite their benign nature. Non-functioning pituitary adenomas (NFPAs) account for 30% of pituitary tumors (2, 3). Although surgery is the primary therapeutic modality, NFPAs frequently have supra- or parasellar extension, owing to which total resection of the tumor is often not possible. Residual tumor regrowth has been reported in as high as 12–64% of patients over a duration of 5 to 15 years (4, 5). True recurrence, in patients with no visible tumor remnant post-surgery, has also been noted in nearly 16% of patients (5). These lines of evidence point towards the unmet knowledge gap in the determinants of recurrence, other than radiological parameters, for NFPAs. Further, the lack of circulating biomarkers and complex tumor pathology of NFPAs act as barriers in delineating the subset of patients at high risk of recurrence. Identification of good prognostic markers of recurrence can not only enable patient stratification for surveillance but also be exploited for their therapeutic potential.

Epidermal growth factor receptor (EGFR) is a transmembrane glycoprotein belonging to the HER family of receptor tyrosine kinases, which promotes multiple signaling cascades for cellular survival, such as RAS/MAPK, PI3K/Akt, Stat, and Src (6). While overexpression of cytoplasmic EGFR (EGFR-C) has been extensively studied as an important molecular target in oncology (7, 8), there have been recent reports stating the importance of nuclear EGFR (EGFR-N) from a similar perspective. EGFR-N regulates target genes involved in cell proliferation and angiogenesis with definite prognostic significance in terms of tumor progression and worse overall survival, especially in breast, ovarian, and lung cancers (9–13). Nuclear EGFR may act as a transcription factor, directly induce phosphorylation of proliferating cell nuclear antigen phosphorylation and confer resistance to radiotherapy (14–17). EGFR activation, resulting in either from mutation or ligand/receptor overexpression, is therefore associated with a variety of human cancers (18).

In the pituitary, EGF acts as a cell growth factor and also directly induces prolactin synthesis (19). Both neoplastic and normal pituitary tissues express EGFR and phosphorylated EGFR [pEGFR] and overall, NFPAs have higher EGFR, pEGFR expression than functional adenomas. Protein phosphorylation is an important post-translational modification characterized by the addition of phosphorus groups to serine, threonine, or tyrosine residues catalyzed by kinase enzymes, resulting in altered protein functions. Characterization of phosphoproteins has directly aided the discovery of protein kinase inhibitors as therapeutic targets in oncology. In the context of the pituitary, the differential expression between normal and neoplastic pituitary tissue is even more amplified when pEGFR expression alone is taken into account as normal pituitary samples do not express this protein. Therefore, phosphoproteins in tumors deserve investigation. Although approximately 60% of pituitary adenomas, including ACTH-secreting adenomas (40%–80%) express EGFR (20–23), the role of the receptor in tumorigenesis remains under-investigated. There are a few reports suggesting high EGFR as a late event in pituitary tumorigenesis and as a marker of aggressiveness in a subset of tumors (21, 22). But the association with disease recurrence, especially in NFPAs, is not known. In our previous mass-spectrometry based study of global phosphoproteomics in NFPAs, we identified 2.6-fold hyperphosphorylation of EGFR at threonine 693 position (pEGFR T693) in recurrent NFPAs (24). The present study was aimed to validate the IHC expression of pEGFR T693 in a large monocentric cohort of NFPAs and correlate with clinical outcomes including recurrence during follow-up.

## Materials and Methods

### Subjects

Over this period (2003-2015), there were a total of 1011 patients with NFPAs managed at the multidisciplinary pituitary clinic, PGIMER, Chandigarh. Of these, patients who underwent adenomectomy by the microscopic trans-sphenoidal route (TSS) and had a minimum of atleast 60 months of follow-up (n=102) were included in the study. Tissue microarray (TMA) was constructed using these NFPA samples obtained from archived samples/blocks at the institute. The specimens were classified into two groups, namely recurrent and non-recurrent, based on radiological surveillance of the patients following TSS. Patients were followed-up post-surgery Controls used were normal pituitary glands obtained from autopsy samples within 3 hours of death (n=5). Both NFPAs and control samples were embedded in paraffin wax to make archival blocks for TMA. The study was approved by the Institute Ethics Committee PGIMER (NK/1790/PhD/6957). Before enrollment, written informed consent was obtained from all patients. All procedures performed in studies involving human participants were in accordance with the Helsinki declaration or comparable ethical standards.

All the cases met the following criteria: i) each paraffin block contained a sufficiently sized specimen to enable the construction of TMAs; ii) no radiation therapy was administered before surgery; iii) complete clinical information, including the endocrinological evaluation and imaging data, was available for a minimum of 60 months.

### Immunohistochemical Analysis (IHC)

The tumor content and quality of all TMA slides were evaluated with hematoxylin and eosin (H&E) staining. NFPAs were classified as the following: gonadotropinomas, silent adenomas or null cell adenomas. Null cell adenomas were defined if they were negative for all 6 anterior pituitary hormones and all 3 transcription factors (Pit1, SF1, T-Pit). Primary antibody for pEGFR T693 (1:50, cat. no. ab75980, Abcam, UK) and EGFR (1:100, ab52894Abcam, UK) were used. Slides were scored for staining positivity (number of cells) and intensity of nuclear pEGFR T693. The intensity of staining for pEGFR T693 was scored as follows: 0, no; 1, weak; 2, moderate, and 3, high intensity (25). The number and distribution of positively stained cells were scored on a scale of 0–100%. A composite h-score was calculated as the product of staining intensity and distribution scores.

### Immunofluorescence

The tissue sections were incubated with primary antibodies [pEGFR T693 (1:50, cat. no. ab75980, Abcam, UK) and EGFR (1:100, ab52894Abcam, UK)]. After washing, slides were incubated with fluorochrome conjugated secondary antibodies. Cell nuclei were stained with 4′,6-diamidino-2-phenylindole (DAPI) and visualised under fluorescence microscope (Evos, Thermo Fisher Scientific, Waltham, MA USA).

### Immunoblots

A total of 30 μg equivalent amount of protein per sample were loaded on 10% SDS–PAGE gel and transferred to nitrocellulose membranes for further processing. The membrane was blocked with 5% bovine serum albumin (BSA) for 1 hour at room temperature, followed by overnight incubation at 4°C with the primary antibodies [pEGFR T693 (1:300, cat. no. ab75980, Abcam, UK)]. Membranes were incubated with appropriate peroxidase-conjugate secondary antibodies (Santa Cruz, USA, 1:3000) and bands were visualized by the enhanced chemiluminescence (ECL) method (BioRad, USA).

### Follow-Up Protocol

All patients were diagnosed based on pre-operative sellar MRI and postoperative histopathology. Trouillas classification was used to grade the tumors based on invasiveness and proliferation (26). The follow-up data for each patient was obtained at 6-month intervals for the first 2 years and then annually. Tumor recurrence was investigated by serial MRI scans of the sellar region performed following surgery, or in the event of recurrence of clinical symptoms, whichever was earlier. Recurrence was defined as the presence of a new tumor in patients with total resection and no discernible residue following TSS, or evidence of regrowth using increment criteria of at least 20% in tumor volume or 2mm in any dimension of an incompletely resected tumor on serial postoperative MRI scans.

### Statistical Analysis

Analyses were performed using SPSS software, version 17.0 (SPSS, Inc., Chicago, IL, USA). Student t-test was used to determine the significance of the association between pEGFR T693 expression, and clinical parameters that were associated with aggressiveness and recurrence. Variables demonstrating a significant association with NFPA recurrence on univariate analysis were subjected to multivariate analysis. Two-tailed p<0.05 was considered to be significant. Survival rates were determined using Cox proportional hazards model analysis.

## Results

### Baseline Characteristics

There were a total of 102 patients with histopathologically proven NFPAs during the study period, for whom follow-up of at least 60 months was also available (**Supplementary Figure 1**). Demographic and clinicopathological parameters of the cohort are summarized in **Table 1**. The mean age of the cohort was 50 ± 11.0 years (range 24-75 years) at presentation with a male preponderance (males 63.7% *vs* females 36.3%). All patients had macroadenomas with a median tumor volume of 14000mm^3^. There were 47 giant adenomas. Recurrence was noted in 46% of patients at a median duration of 123 months (range 60-213months) of follow-up. IHC showed a positive nuclear pEGFR T693 expression in 88% (90/102) of the specimens. Of the 90 positive specimens, 50 showed weak staining, 37 moderate, and 3 high-intensity staining.

**Table 1:** Baseline clinical, radiological and histopathological characteristics of the cohort with composite h-scores stratified on the basis of demographic characteristics.

Parameter		pEGFR T693 h-score		p-value
		Range (Min-Max)	Mean ± SD	
**Age**	<30 years (n=11)	7.05-201.1	82.2 ± 17.1	0.67
	30-50 years (n=69)	11.5-227.4	94.3 ± 17.1	
	>50 years (n=22)	41.8-166.6	94.0 ± 7.0	
**Gender**	Female (n=37)	13.8-227.4	93.9 ± 7.5	0.99
	Male (n=65)	7.0-209.3	93.1 ± 5.0	
**Tumor diameter**	Giant (n=47)	7.0-227.4	90.7 ± 6.9	0.57
	Macro (n=55)	11.5-209.3	95.6 ± 5.0	
**Tumor volume**	Above median (14000mm^3^)	7.0-209.3	92.3 ± 5.3	0.88
	Below median (14000mm^3^)	11.5-227.4	92.3 ± 6.6	
**Tumor extension**	Intrasellar (n=30)	49.9-209.3	93.6 ± 7.3	0.47
	SSE (n=46)	11.5-201.1	86.3 ± 5.7	
	SSE+PSE (n=16)	41-166.6	96.0 ± 9.8	
	SSE+PSE+ISE (n=6)	7.0-227.4	119.3 ± 30.8	
	SSE+ISE (n=4)	64.4-158.2	94.7 ± 21.7	
**Clinico-pathological grading by Trouillas et al**	1a (n=44)	11.5-201.1	81.8 ± 5.8	0.05
	1b (n=7)	101-158	130.7 ± 9.2	
	2a (n=43)	7.0-227.4	95.6 ± 6.2	
	2b (n=8)	13.8-178.8	128.4 ± 10.7	
	1a+2a (n=87)	7.0-227.4	89.1 ± 4.3	0.02*
	1b+2b (n=15)	13.8-178.8	116.2 ± 11.0	
**IHC**	Silent Adenomas (n=21)	32.7-201.1	105.1 ± 9.0	0.15
	Gonadotropinomas (n=72)	13.8-227.4	91.1 ± 4.8	
	Null Cell adenomas (n=9)	7.0-141	73.6 ± 14.3	
**Recurrence**	Recurrent (n=47)	34.6-227.4	117 ± 6.0	<0.0001****
	Non-recurrent (n=55)	7.0-144.4	71.2 ± 3.7	

Data are presented as range or mean ± SD.

SSE, suprasellar extension; PSE, parasellar extension; ISE, infrasellar extension; IHC, immunohistochemistry. *significant, ****highly significant. The bold values refer to significant parameters.

### Age and Gender

Patients were grouped into three age classes: younger than 30 years (I), 30-50 years (II), and older than 50 years (III). The rates of recurrence were not significantly different between the 3 groups (45% *vs* 46% *vs* 45%, p=0.85). In group I (n=11), 7 patients showed positive nuclear pEGFR T693 expression with a mean h-score of 82.2 ± 17.1 (range 7.0-201.1). In group II (n=69), 62 patients showed positive IHC with a mean h-score of 94.3 ± 17.1 (range 11.5 to 227.4). In group III (n=22), 21 patients showed positive IHC with a mean h-score of 94.0 ± 7.0 (range 41.8-166.6). However, there was no significant difference between h-scores of different age groups (p=0.67) (**Supplementary Figure 2A**). The mean h-scores of age group II (p<0.0001) and III (p=0.0005) showed a significant difference between recurrent and non-recurrent tumors.

Male preponderance was noted in both recurrent and non-recurrent groups (65.9% *vs* 63.6%, p = 0.51). Overall, 86.2% (56/65) of the tumors of male patients and 91% (34/37) of the women were positive for pEGFR T693. Among male patients, 47.6% tumors (n=31) were weakly positive, 36.9% (n=24) moderate, and 1.5% (n=1) strongly positive. In females, pEGFR T693 staining scores classified 52.7% (n=19) as weakly positive, 33.3% (n=12) as moderate and 5.5% (n=2) as strongly positive. There was no significant difference between mean h-scores of males and females (93.1 ± 5.0 *vs* 93.9 ± 7.5, p=0.99). (**Supplementary Figure 2B**).

### Tumor Subtypes

Patients were classified based on the tumor subtype as gonadotropinomas, null cell adenomas, or silent adenomas. The recurrence rate was 71.4%, 38.8, and 44.4% in silent adenomas, gonadotropinomas, and null cell adenomas respectively. The majority of the gonadotropinomas (88.9%) (39 were weak, 23 moderate and 2 were strong positive) showed positive staining (mean h-score 91.8 ± 4.8) while 77% null cell adenomas (5 weak, 2 moderate positive) showed positive staining (mean h-score 73.6 ± 14.3) for pEGFR T693. Among silent adenomas, 90% showed (19/21) positive staining with a mean h-score of 105.1 ± 9.0. There was no significant difference in h-scores between the groups (p=0.157) (**Supplementary Figure 2C**).

### Tumor Grade

High Ki67 (>3%) was found in similar proportion of patients in both recurrent and non-recurrent groups (19.1% *vs* 18.2%, p=0.91). NFPAs were classified based on the proposed clinicopathological classification by Trouillas et al. Eighty-four percent of tumors (n=37) with grade 1a (non-invasive, non-proliferative) were positive for pEGFR with a mean h-score of 81.8 ± 5.8 (range 11.5-201). There were 27 weakly positive, 9 moderate, and 1 strongly positive tumor. All grade 1b (non-invasive, proliferative) tumors (n=7) were positive for nuclear pEGFR with a mean h-score of 130.7 ± 9.2 (range 101-158). Ninety % (39/43) of grade 2a (invasive, non-proliferative) tumors were positive for nuclear pEGFR T693 with mean h-score 95.6 ± 6.2 (range 7.0-227.4). All patients with grade 2b (invasive, proliferative) NFPAs were positive with a mean h-score of 128.4 ± 10.7 (range 13.8–178.8), one was weak positive while five were moderate positive. There was no significant difference between the mean h-scores across the tumor grades (p=0.05) (**Supplementary Figure 2D**). Proliferative tumors (1b+2b) had significantly higher mean h-scores than non-proliferative (1a+2a) (p=0.02) (**Supplementary Figure 2E**).

### Tumor Size and Volume

There were 47 giant tumors, of which 38 (80%) were positive for nuclear pEGFR with a mean h-score of 90.7 ± 6.9. Ninety-two percent of the macroadenomas (51/55) showed positivity with a mean h-score of 95.6± 5.0. Statistically, there was no difference between the number of giant NFPAs or macroadenomas staining positive for pEGFR (p= 0.57) (**Supplementary Figure 3A**). There was no correlation between tumor size, volume, or Knosp grade and h-score of pEGFR (**Supplementary Figures 3A–E**).

### Recurrence

**Figure 1:**
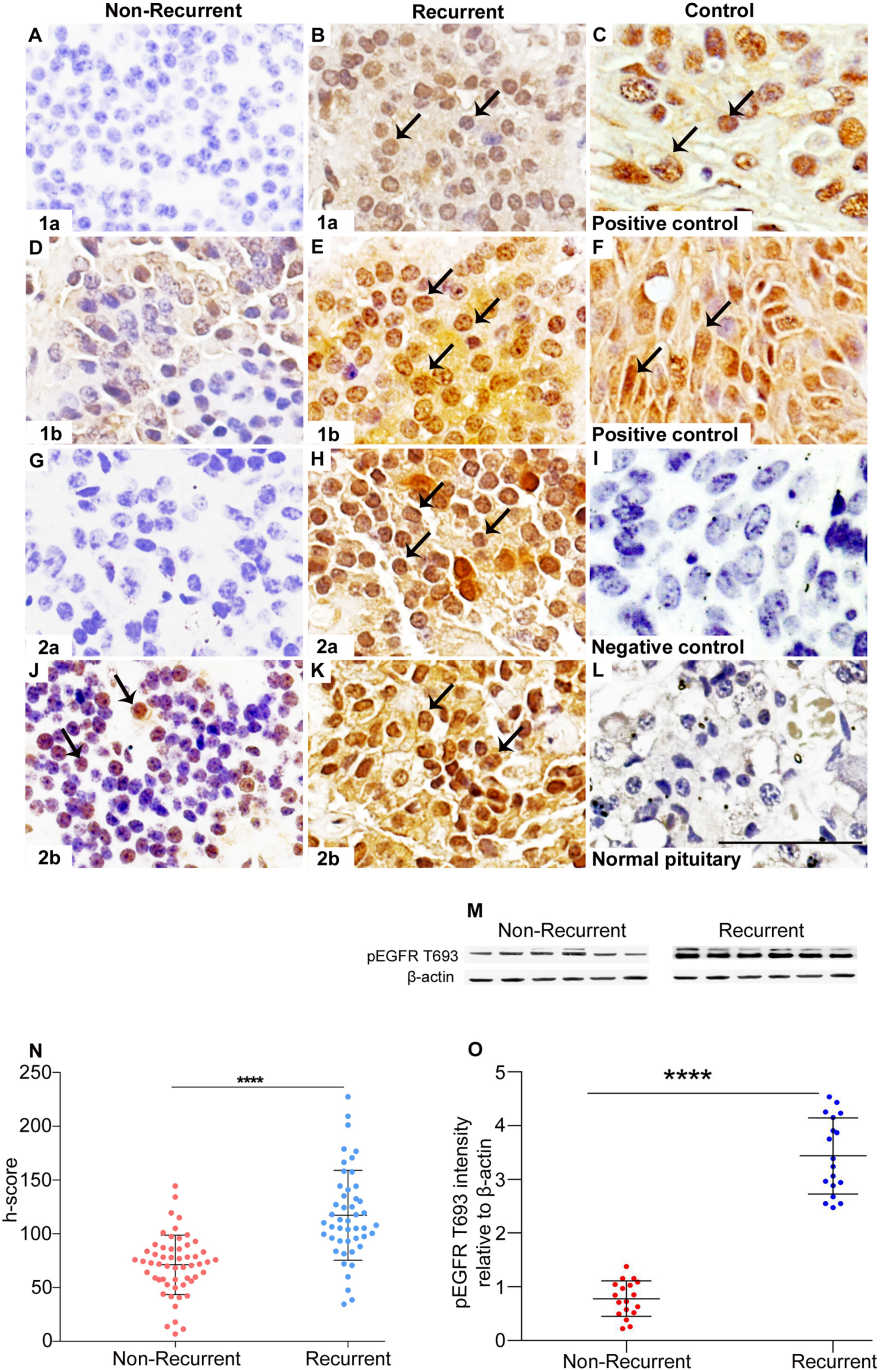
Overexpression of pEGFR T693 in recurrent NFPAs. Immunohistochemistry of pEGFR T693 showed overexpression in recurrent NFPAs **(B, E, H, K)** as compared to non-recurrent NFPAs **(A, D, G, J)**. pEGFR T693 showed strong nuclear positivity (brown) marked by black arrows. Breast cancer **(C)** and cervical cancer **(F)** were used as the positive controls. No staining was observed in the negative control **(I)** and normal pituitary **(L)**. Western blots of representative tumor samples are depicted in **(M)**. The h-score (product of the number of cells with positive staining and staining intensity) showed statistically significant overexpression of pEGFR T693 in recurrent NFPAs as compared to non-recurrent (p < 0.0001) **(N)**. Quantification of the blots **(O)** show overexpression of pEGFR T693 in representative tumor samples of 18 recurrent NFPAs as compared to 18 non-recurrent ones. Magnification 400x. ****highly significant.

**Figure 2:**
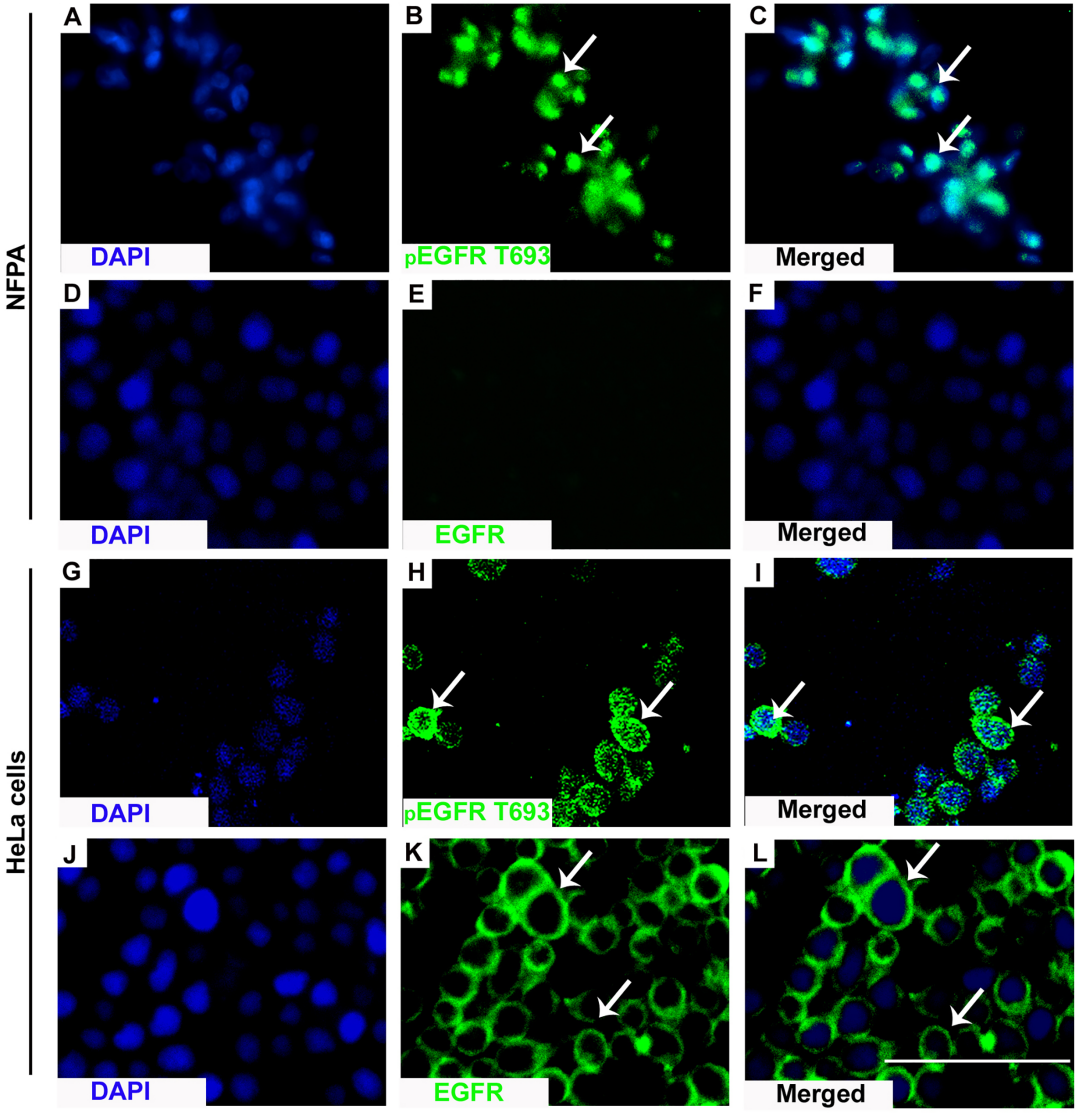
Nuclear localization of pEGFR T693. IF showed nuclear localization of pEGFR T693 in NFPAs **(A–C)** and HeLa cells as the positive control **(G–I)**. In HeLa cells, EGFR showed membranous positivity **(J–L)** while no EGFR expression was observed in NFPAs **(D–F)**. EGFR and pEGFR T693 are shown by green while the nucleus is represented as counterstaining by DAPI (blue). Magnification 400x.

**Figure 3:**
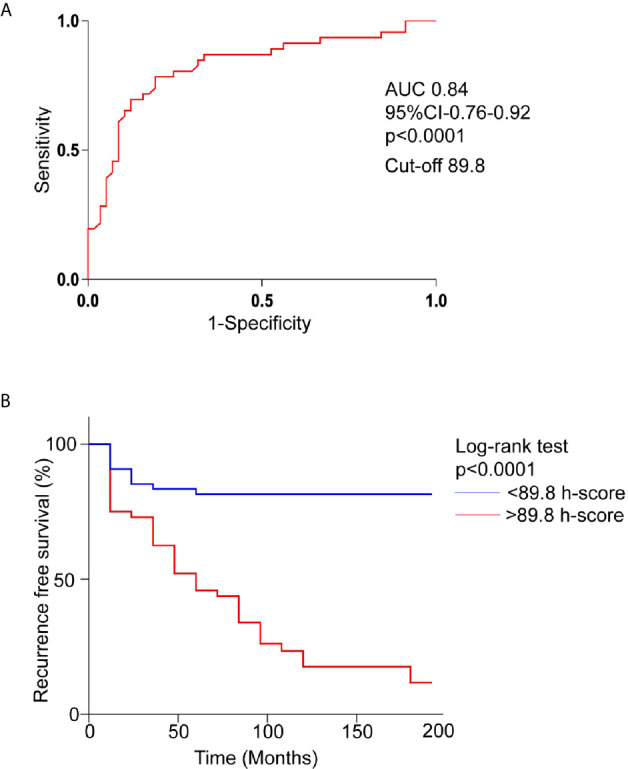
Overexpression of pEGFR T693 as an independent prognostic factor of recurrence in NFPAs. **(A)** Receptor operating characteristics curve (ROC) for prediction of recurrence based on pEGFR T693 h-score showed an h-score cutoff of 89.8 (80% sensitivity, 78% specificity) and an area under curve of 0.84 (p < 0.0001) indicating pEGFR T693 as a good biomarker of recurrence **(B)** Kaplan–Meier recurrence-free survival of the patients after surgery for NFPAs (n=102) stratified according to the cut-off level of h-score 89.8. High levels of pEGFR T693 as an independent predictor of recurrence (p < 0.0001). The red line indicates patients with an h-score higher than 89.8 and the blue line indicates an h-score less than 89.8.

Positive pEGFR IHC was found in a significantly more number of subjects with recurrent than non-recurrent tumors (95.7% *vs* 81%, p=0.02). In the 55 non-recurrent cases, 81% (n=45) were positive for pEGFR with 7 demonstrating moderate and 38 low positive staining. Among the 47 recurrent NFPAs, 95.7% (n=45) were positive for pEGFR T693 with 3 showing strong, 30 moderate, and 12 low positive staining (**Figure 1**). Further analysis was performed using clinically relevant staining intensity of nuclear pEGFR T693, defined as moderate or strong staining. This showed a significantly greater proportion of patients in the recurrent NFPA group (**Figure 1**) than the non-recurrent group (73.3 *vs* 15.6%, p<0.0001). Non-specific antibody binding was excluded by using positive controls (breast cancer (**Figure 1C**), cervical cancer (**Figure 1F**), and omission of primary antibody in the above-mentioned tissue was used as negative control (**Figure 1I**). Normal pituitary controls obtained from autopsy samples were also found to be negative for pEGFR T693 immunostaining (**Figure 1L**). The hyperphosphorylation of pEGFR T693 in recurrent NFPAs was also confirmed by Western blot (**Figure 1M**). Recurrent NFPAs also had significantly higher h-scores than non-recurrent tumors (117 ± 6.0 *vs* 71.2 ± 3.7, p<0.0001) (**Figure 1N**). The nuclear positivity of pEGFR T693 was confirmed by immunofluorescence in these tumors (**Figures 2A–C** and using HeLa cell lines as positive control, **Figures 2G–I**). Total EGFR showed membranous positivity in Hela cells (positive control) (**Figure 2J–L**) while no EGFR expression has been observed in NFPA (**Figures 2D–F**). Immunohistochemistry showed the absence of EGFR expression in 96% of NFPAs (**Supplementary Figure 4**). There was a significant correlation between the h-score of pEGFR T693 and the recurrence of NFPAs (r=0.554, p=0.000) (**Table 2**). Receiver operating curve (ROC) analysis for pEGFR h-scores used in differentiating recurrent from non-recurrent tumors revealed a cutoff of 89.8 as significantly predicting recurrence (sensitivity 80%, specificity 78%, AUC 0.84, 95% CI 0.76-0.92, p < 0.0001) (**Figure 3A**).

**Table 2:** Pearson’s correlation between tumor characteristics and pEGFR T693 h-score.

Parameter	Pearson’s r	p value
Age	0.04	0.66
Gender	0.001	0.99
Tumor Diameter	0.05	0.57
Tumor Volume	0.001	0.99
Tumor Extension	0.089	0.37
**Clinico-pathological grading by Trouillas et al.**	**0.225**	0.03*
Immunohistochemistry	-0.194	0.05
**Recurrence**	**0.554**	0****

*significant ****highly significant. The bold values refer to significant parameters.

Univariate analysis showed positive pEGFR T693 staining (p<0.0001) and tumor grade (p=0.02) to be significantly associated with recurrence, while other clinicopathological parameters were not associated with NFPAs recurrence (**Table 1**). A significant association between pEGFR T693 expression and recurrence (HR=4.9, CI 2.8-8.8, P<0.0001) was observed. The association of tumor grade as per the recently proposed clinicopathological classification (26) with recurrence of NFPAs in our study was 1.7 (CI-0.7-3.9, p=0.10).

### Recurrence-Free Survival Analysis

During follow-up, 46% patients (n=47) experienced tumor recurrence. Patients were divided into two groups based on the h-score cut-off of 89.8 (**Figure 3A**). In the Kaplan-Meier survival curve comparing the 17-year overall recurrence-free survival, patients with h-score less than 89.8 had higher overall recurrence-free survival compared to the patients with higher h-score (p<0.0001) (**Figure 3B**).

## Discussion

The current study is the first, to the best of our knowledge, to investigate the association of phosphorylated EGFR (pEGFR T693) with the recurrence of NFPAs. We demonstrated that pEGFR T693 expression (both intensity and percentage of cells) was increased in the recurrent NFPAs compared with the non-recurrent NFPAs. We also found a greater risk conferred by pEGFR T693 for NFPA recurrence as compared to conventionally used parameters like tumor grade (a composite measure of invasiveness and proliferation). Further, at a median period of follow-up of 72 months, 46.1% of patients experienced tumor recurrence, significantly predicted by the identified cutoff of h-score (89.8) for pEGFR T693. These findings suggest the potential utility of nuclear pEGFR T693 in predicting recurrence in NFPAs. In association with tumor grade, pEGFR T693 can possibly be used for identifying the subset of patients with a high risk of recurrence, to improve their surveillance frequency, and initiate early management in case of clinically significant tumor recurrence.

NFPAs are characterized by lower rates of remission than functioning pituitary adenomas (27). Recurrence or regrowth of residual tumor has been observed in up to 15 to 66% of patients treated with surgery alone and up to 28% with the use of adjuvant radiotherapy over a median follow-up duration of 5.9 years (28). Even when patients with clinically significant recurrence requiring re-intervention are considered, the rates are as high as 20% (29). The higher recurrence rate of NFPAs compared to pituitary tumors overall suggests that multiple factors may be responsible for this biological behavior and not tumor size alone. There is also a rare but existent risk of carcinomatous transformation in patients with recurrent and/or invasive NFPAs (30). While functional pituitary adenomas can be easily followed up for recurrence using biomarkers, there is no similar parameter to predict recurrence in NFPAs, thereby delaying early recognition and timely management.

Factors predicting recurrence in NFPAs have not been proven conclusively. In a meta-analysis, clinical parameters like age, gender, tumor size, invasion, and proliferation were found to be significant in some studies but not in others (27, 31, 32). In more recent studies, the Trouillas classification of tumor grading based on invasion and proliferation was found to predict recurrence with the highest-grade lesions (2b invasive and proliferative) harboring an almost 9 times higher risk of recurrence than the lowest grade lesions (1a invasive) (33). In other reports, MRI texture analysis parameters enabled the prediction of recurrence even after adjusting for other factors like age, Ki67, and completeness of resection (34). The tumor subtype has been reported to be associated with higher recurrence in some studies but not in others (35–37). Overall, there exist a lacuna inaccurate predictive factors that could help stratify patients based on low or high risk of recurrence or progression. In the context of a notably high rate of recurrence and lack of suitable biomarkers of hypersecretion to identify recurrence, this remains enigmatic.

Microarray analysis studies are limited in the context of recurrence prediction of NFPAs. In a small study, circulating RNAs, especially the circ RNA 102597 was found to predict recurrence using these assays in conjunction with other described factors including Ki67 (38, 39). β-catenin has been found to predict recurrence in another study (40). Protein phosphorylation, one of the most important post-translational modification processes, enables enrichment of protein diversity. The total number of proteoforms significantly exceeds the number of transcripts and genes in normal individuals in the hierarchy of omics, it is ultimately the proteomics of a cell that decides its functions and fate. Hence, evidence on phosphoproteomics, though less well characterized, holds the promise of being able to clarify the still elusive molecular mechanisms involved in the genesis and progression of NFPAs. Hence, it can be contemplated that phosphorylation-mediated cellular events that occur as part of post-translational modification of proteins have a putative role in regrowth of remnant tumor or recurrence in case of no discernible residue post-surgery. In line with this postulation, there is some evidence to suggest that phosphorylated AMPK/ATF2 is involved in tumorigenesis in GH secreting pituitary adenomas (41). However, knowledge about phosphoproteome profiles of NFPAs is limited (42). In the context of NFPAs, the pursuit of biomarkers that enable the prediction of recurrence and can be employed as potential drug targets is certainly encouraging in the current era of personalized medicine.

EGFR plays an important role in the progression of many types of cancer by activation of mitogenic pathways, altering cell cycle progression, and by downregulating tumor suppressor genes. Nearly two decades ago, two independent reports documented nuclear EGFR status in bladder and cervical cancers using IHC. While these early studies were the first to quantitate nuclear EGFR in patient tumors, they did not examine the prognostic value of nuclear EGFR expression. The Thr 693 phosphorylation of EGFR by p38 leads to its internalization. Internalized EGFR can form signaling complexes in endosomes, which may trigger different signals than the membrane-localized receptor (43). The nuclear counterpart of EGFR appears to be the full-length receptor and likely, in the phosphorylated form, as shown by many studies. In other studies, EGFR/ERK signaling pathway has been found to mediate cell migration, invasion, and proliferation (44).

Among pituitary adenomas, EGFR has been reasonably well studied in corticotropinomas, where IHC positivity in nearly 55% of tumors has been documented (45). Further, the degree of expression in these tumors was found to be much greater than normal pituitary samples and it was also found to be correlated with tumor burden in terms of ACTH and cortisol. In that study, pEGFR was found to be significantly associated with tumor recurrence but there was no association with age, gender or duration of symptoms, or tumor size suggesting the utility of EGFR as a biomarker for predicting recurrence. However, the study was silent regarding the exact site of the phosphorylation. Further, in corticotropinomas, EGFR overexpression is a consequence of 14-3-3 USP8 mutations, which are, in fact, quite specific to this tumor subtype. Thus, the pEGFR found in NFPAs, with the unique phosphorylation site at Thr 693, maybe a distinctive step in the molecular pathogenesis of NFPAs that determines their recurrence. Further, available evidence suggests almost a three-fold higher pEGFR expression in NFPAs than functional adenomas (21). EGFR has been identified as a transcriptional co-activator for several cancer-promoting genes, including cyclin D1, nitric oxide synthase (iNOS), and cyclooxygenase-2 (COX-2) (46, 47). Cyclin D1 has a bearing on cell proliferation, recurrence, and aggressive behavior of pituitary tumors (48). Overexpression of cyclin D1 in NFPAs maybe secondary to the molecular aberration of nuclear translocation of EGFR due to phosphorylation at Thr 693 site.

The translational benefit offered by the current study can be extended to future studies using targeted therapy (pEGFR 693 antagonist) both *in-vitro* and in clinical settings to restrain the growth and recurrence of NFPAs. Results from the current study implicate nuclear EGFR in the etiology of recurrent NFPAs and patients with IHC positive for pEGFR T693 should be followed up more closely. This specific marker holds the dual advantage of predicting recurrence as well as being targeted for therapy. It is expected to be of maximum benefit in tumors with a high expression of this receptor. Usage of drugs like gefitinib and erlotinib for aggressive ACTH producing pituitary adenomas (49) and recently, lapatinib for aggressive prolactinomas (50) has produced encouraging results, but similar therapies have not hitherto been attempted in recurrent NFPAs. Further, assessment of synergistic efficacy of this treatment modality in conjunction with radiotherapy could also be a potent therapeutic modality for patients with NFPAs, which warrants investigation in future studies.

## Conclusion

The current study provides evidence that nuclear pEGFR T693 expression is significantly higher in recurrent than non-recurrent NFPAs. Prediction of recurrence was higher with phosphorylated EGFR than tumor grade and there was no association with baseline tumor size. pEGFR T693 is a potential biomarker to predict recurrence in NFPAs.

## Data Availability Statement

The raw data supporting the conclusions of this article will be made available by the authors, without undue reservation.

## Ethics Statement

The studies involving human participants were reviewed and approved by Institute Ethics Committee, PGIMER, Chandigarh, India. The patients/participants provided their written informed consent to participate in this study.

## Author Contributions

All authors contributed equally to the study and have read and approved the final version of the manuscript.

## Funding

We thank the Department of Biotechnology, Government of India for research support [6242-P109/RGCB/PMD/DBT/KNMJ/2015] to the PGIMER. We thank the Council of Scientific and Industrial Research, University Grants Commission, and the Government of India for financial support. AR is a recipient of a Senior Research Fellowship from the Council of Scientific and Industrial Research/University Grants Commission, Government of India.

## Conflict of Interest

The authors declare that the research was conducted in the absence of any commercial or financial relationships that could be construed as a potential conflict of interest.

## Supplementary Material

Supplementary Figure 1 Flow diagram of NFPAs recruited in the study.

Supplementary Figure 2 pEGFR T693 overexpressed in proliferative NFPAs. Quantitative h-score of pEGFR T693 immunohistochemistry with clinical parameters **(A–E)** showed no differential expression across age **(A)**, gender **(B)**, immunohistochemistry of hormones **(C)**, and Trouillas classification **(D)**. Proliferative tumors as per Trouillas classification (1b + 2b) showed significantly higher expression of pEGFR T 693 as compared to non-proliferative NFPAs (1a+2a) **(E)**. Age group (A)- 1: < 30 years, 2: 30-50 years, 3: >50 years .SA, silent adenoma; GN, gonadotropinoma; NC, null cell adenoma.

Supplementary Figure 3 NFPAs quantitative h-score of pEGFR T693 immunohistochemistry with tumor size and extension **(A–E)** showed no differential expression across tumor diameter **(A)**, tumor volume **(B)**, suprasellar extension **(C)**, parasellar extension **(D)**, and infrasellar extension **(E)**. The Student’s t-test was used for comparing the groups. SSE-suprasellar extension, PSE-parasellar extension, ISE-infrasellar extension.

Supplementary Figure 4 EGFR expression in NFPAs (n=50). Positive EGFR expression was observed in two patients **(D)** and while others were negative **(E)**. Cervical carcinoma was used as the positive control **(A)** while there was no expression of EGFR in normal pituitary (n=5), Cushing’s (n=10), and negative control. Magnification 400x.
