# Supplementary figures and images for: Phosphorylated EGFR (pEGFR T693) as a Novel Predictor of Recurrence in Non-Functioning Pituitary Adenomas
Source: Front Endocrinol (Lausanne). 2021 Jul 5;12:708111. doi: 10.3389/fendo.2021.708111 (PMC8289705; doi:10.3389/fendo.2021.708111)

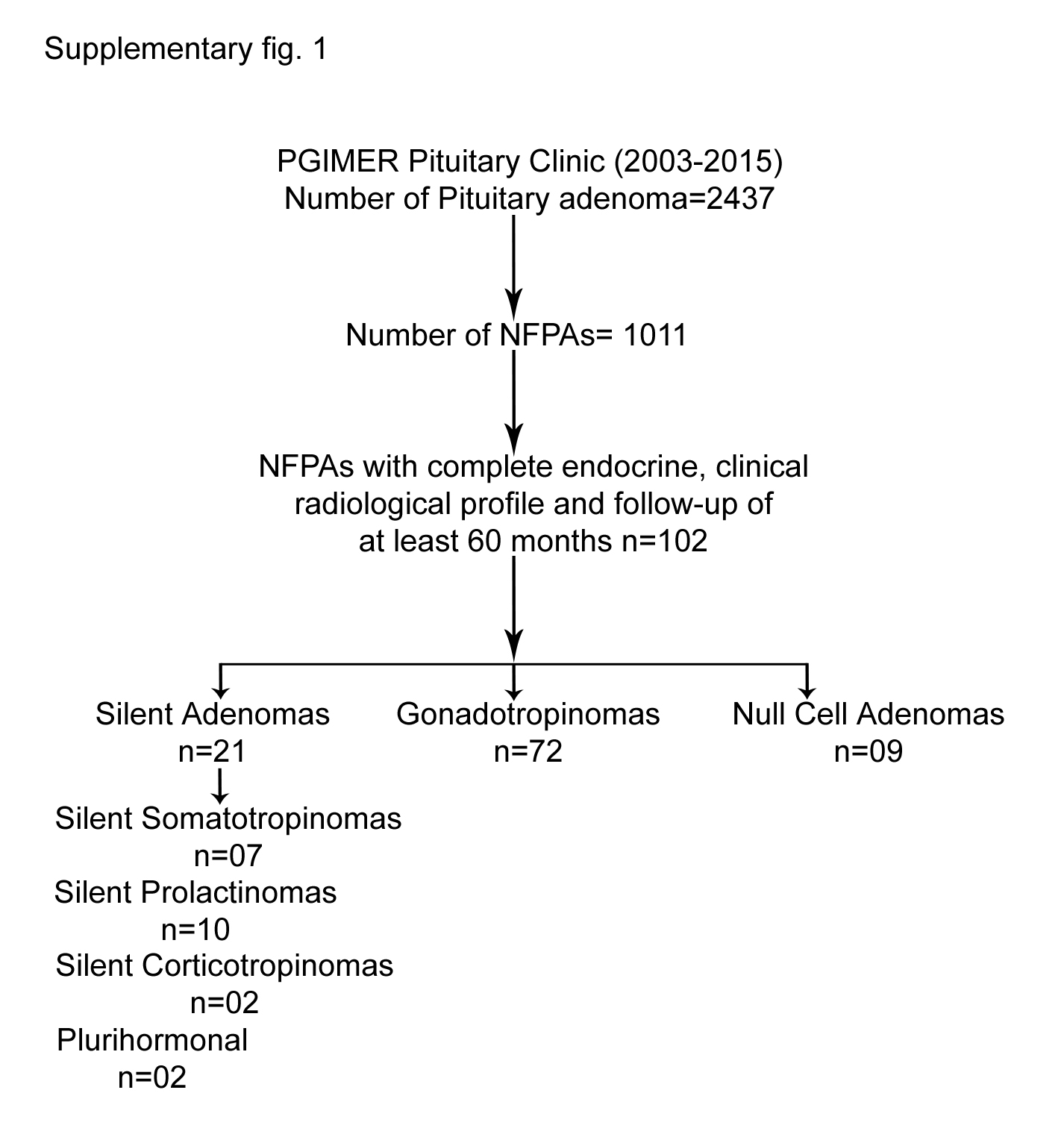

Supplement: Supplementary Figure 1 — Flow diagram of NFPAs recruited in the study. [file Image_1.jpeg]

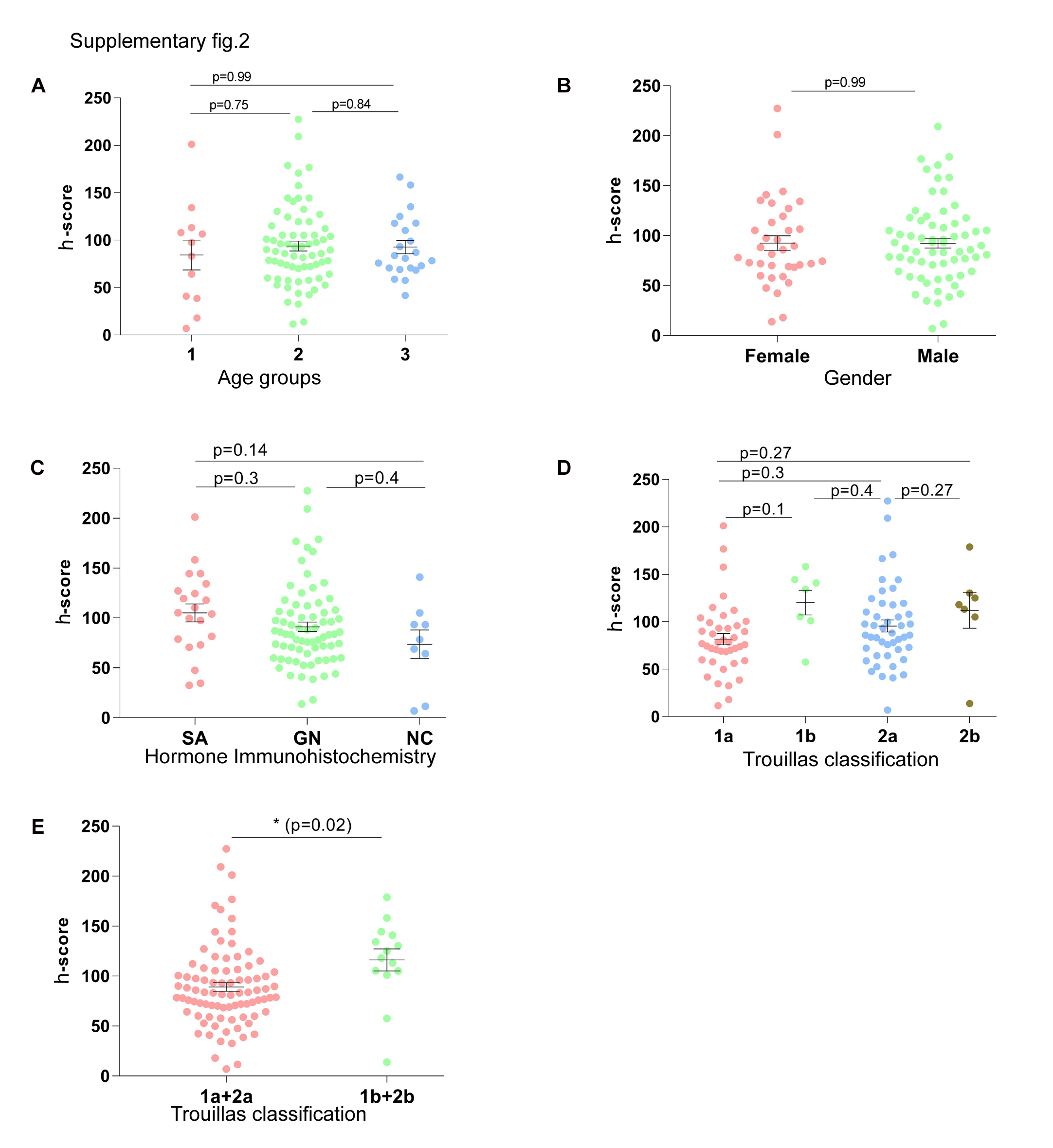

Supplement: Supplementary Figure 2 — pEGFR T693 overexpressed in proliferative NFPAs. Quantitative h-score of pEGFR T693 immunohistochemistry with clinical parameters (A–E) showed no differential expression across age (A), gender (B), immunohistochemistry of hormones (C), and Trouillas classification (D). Proliferative tumors as per Trouillas classification (1b + 2b) showed significantly higher expression of pEGFR T 693 as compared to non-proliferative NFPAs (1a+2a) (E). Age group (A)- 1: < 30 years, 2: 30-50 years, 3: >50 years .SA, silent adenoma; GN, gonadotropinoma; NC, null cell adenoma. [file Image_2.jpg]

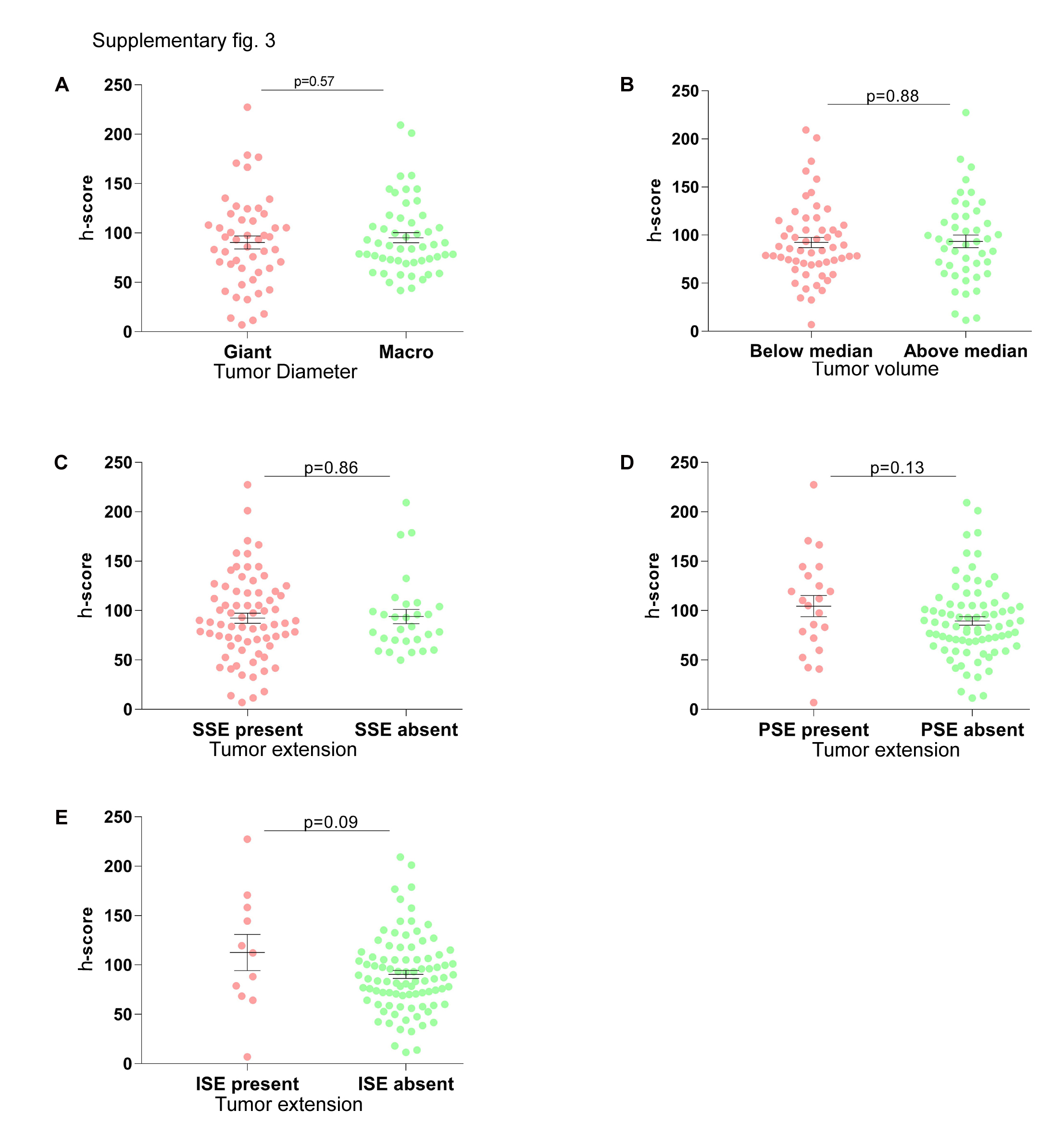

Supplement: Supplementary Figure 3 — NFPAs quantitative h-score of pEGFR T693 immunohistochemistry with tumor size and extension (A–E) showed no differential expression across tumor diameter (A), tumor volume (B), suprasellar extension (C), parasellar extension (D), and infrasellar extension (E). The Student’s t-test was used for comparing the groups. SSE-suprasellar extension, PSE-parasellar extension, ISE-infrasellar extension. [file Image_3.jpg]

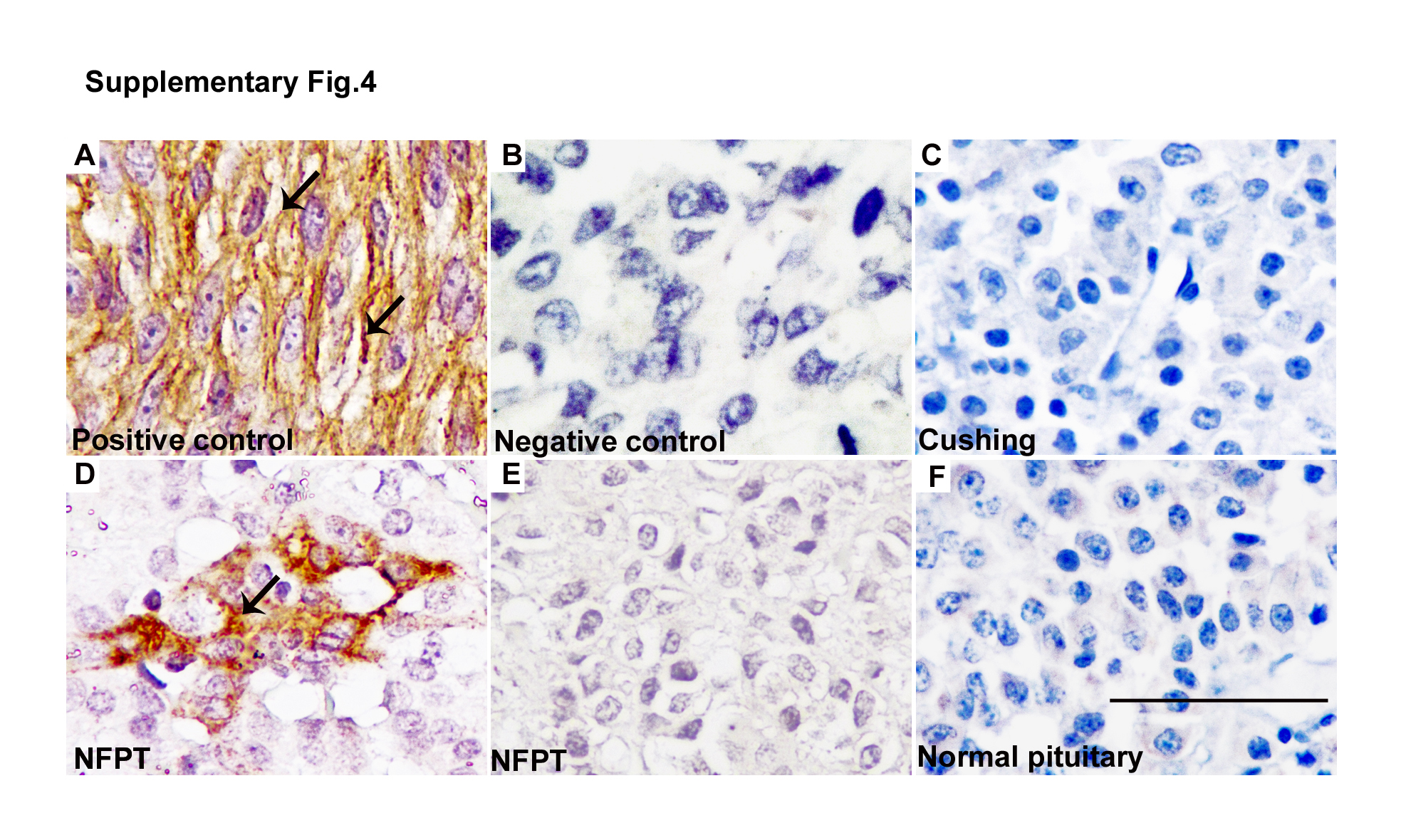

Supplement: Supplementary Figure 4 — EGFR expression in NFPAs (n=50). Positive EGFR expression was observed in two patients (D) and while others were negative (E). Cervical carcinoma was used as the positive control (A) while there was no expression of EGFR in normal pituitary (n=5), Cushing’s (n=10), and negative control. Magnification 400x. [file Image_4.jpg]
